# A Notice of the Grayson Springs

**Published:** 1847-03

**Authors:** Lunsford P. Yandell

**Affiliations:** Louisville


*A Notice of the Grayson Springs.* By LUNSFORD P. YANDELL, M. D.—The present proprietor of the Grayson Springs, in the summer of 1833, learning that quite a number of infirm people had gone to them in the woods, and were encamped about them in tents, was induced to purchase and improve them. It seems that, many years ago, they acquired, by some accident, a reputation for curative virtues, and this they have continued to enjoy. On a visit to the springs last summer, I met with a large company of invalids, and learned from many of them that they had not been disappointed in their hopes of renovated health from the use of the waters. I saw some lodged in hired cottages, near the springs, who were not able to pay for board at a tavern, and had brought with them their beds, provisions, and cooking utensils—a fact which testifies to the strong popular faith in the efficacy of these springs.

The springs, issuing within an acre of ground, are five in number, and, although differing in temperature, and slightly in taste, contain essentially the same ingredients. The Rock spring is the coldest; the Stump spring is so warm as to be unpalatable, and its water is chiefly used to supply the bath-house. The water of the Centre, Moreman, and McAtee springs has a pleasant temperature. Of these the latter is perhaps the favorite fountain. It takes its name from an early visiter, a lady, whose health is said to have been restored by the use of its waters.

The following are the ingredients found in these springs:

Sulphuretted hydrogen,
Carbonic acid,
Carbonate of magnesia,
Carbonate of lime,
Carbonate of iron,
Sulphate of magnesia,
Sulphate of lime.

Sulphuretted hydrogen is the most characteristic constituent of the waters, and, with the carbonic acid, renders them light. The first effect of drinking them is an increase of perspiration ; the bowels are generally gently moved, and the appetite and powers of digestion improved. To generalize, the action of the Grayson springs may be said to be gently tonic, laxative, and alterative.

These springs occur in a different formation from the other noted mineral springs of Kentucky ; as they also differ materially from most of them in their constitution. Sulphate of magnesia is the most prominent ingredient in the Harrodsburg springs, which is recognised by its bitter taste ; and although the same article is found in the Grayson springs it is in too minute a quantity to affect the taste of the water, which is sweetish. The Blue, Bigbone, and Drennon's Licks, the Olympian and Paroquet springs, all abound in common salt, which is absent from the waters of Grayson. They all, except the Olympian, occur in the Blue Limestone, the oldest of our fossiliferous rocks ; the Grayson springs are found in the Carboniferous limestone, immediately below the coal series. The stratum from which they issue is the same in which the Mammoth Cave occurs,—the *Pentremital* or uppermost layer of the most recent of our limestone rocks. In this rock, in the immediate vicinity of the springs, Pentremites, Archimides, and many new and most beautiful species of Encrinites, are found. The locality is one of exceeding interest to the geologist.

*Louisville*, Jan. 26th, 1847. *Western Journal.*

